# Smaller Saami Herding Groups Cooperate More in a Public Goods Experiment

**DOI:** 10.1007/s10745-016-9848-3

**Published:** 2016-09-19

**Authors:** Matthew Gwynfryn Thomas, Marius Warg Næss, Bård-Jørgen Bårdsen, Ruth Mace

**Affiliations:** 1 Department of Anthropology, University College London, 14 Taviton Street, London, WC1H 0BW UK; 2 Norwegian Institute for Nature Research, Fram Centre, Tromsø, 9007 Norway; 3 Norwegian Institute for Cultural Heritage Research, Fram Centre, Tromsø, 9007 Norway

## Introduction

Group living often entails a balance between individual self-interest and benefits to the group as a whole. Situations in which an individual’s vested interests conflict with collective interests are known as social dilemmas (Kollock 1998). More formally, a theoretical game becomes a social dilemma when an equilibrium of dominant strategies leads to worse outcomes for all players compared to a more cooperative but non-equilibrium strategy (Zelmer 2003; Cardenas and Carpenter 2008). For example, arms races, climate change, the Cold War, credit markets, eBay, exploitation of fisheries, irrigation scheduling, overpopulation, pollution, price wars, voting, water supply and welfare states all give rise to social dilemmas (Kollock 1998; Wydick 2008).

Researchers have identified various mutually inclusive routes to solving social dilemmas, including interacting with kin and/or cooperative individuals, communication, coordination, exclusion, institutions, leadership, legislation, mobility, monitoring, parcelling out cooperation or access to resources, partner choice, partner control, policing, punishment, repeated reciprocal interactions, rewards, sanctions, and social norms (Trivers 2005; West *et al.* 2007; Levin 2014; Raihani and Bshary 2015).

Social dilemmas pervade the pastoralist way of life. Individual herders must balance their interests (e.g., generating income and managing the inherent risks of pastoralism) with the interests of their herding group and the wider community facing similar challenges (Næss *et al.* 2012; Næss and Bårdsen 2015). Pastoralists such as Saami reindeer herders in Norway face social dilemmas across a range of scales and have a variety of individual and collective strategies for solving them.

### Social Dilemmas Facing Individual Herders and Herding Groups

Individual herders make husbandry decisions regarding the management of their herd, including shaping the herd’s phenotypic structure and slaughter strategies (Paine 1994). Decisions to slaughter depend not only on an individual’s herd size but also positively correlate with the slaughtering decisions of neighbours; herders do not want to be in a position where they reduce their herds more than their neighbours, thus risking a competitive disadvantage (Næss *et al.* 2012).

One way individual herders can choose to cooperate with others is by investing labour in common projects. Labour investment can be understood as akin to provisioning public goods, in the sense that everybody benefits from the labour of those who help with herding tasks such as gathering reindeer for spring corrals.

Saami pastoralists organise themselves into cooperative herding groups known as *siidas*, which are the focal points of cooperation (Thomas *et al.* 2015). The siida is a collective action group tied to an area of pastureland. Herders belong to a larger summer siida and a smaller winter siida (with some also being part of autumn siidas), meaning they cooperate with different people throughout the year in groups of differing sizes.

Cooperation within siidas takes many forms and fulfils many purposes. The size of the siida affects the nature of collective action within the group as well as the group’s productivity/long-term viability. Siidas with more members are likely to have larger herds (Næss *et al.* 2009), which helps to prevent land-grabs from competing groups. Although this is less of a worry given the current trend towards privatised and fenced land, pastoralists still face the task of preventing unwanted encroachment.

Similarly, herders must work together to protect their livestock from predators, not all of which can be legally hunted (this is especially relevant for the one siida in our study area whose summer pasture is located in a national park). Collective labour is also needed for seasonal activities such as harvest, earmarking calves and splitting up herds before migrations. Contribution problems such as provisioning public goods (e.g., deciding whether to invest time and energy in collective action) are likely to exist in concert with consumption problems involving how to best utilise common-pool resources.

### Multi-Scale Social Dilemmas

The benefits of cooperative acts can extend beyond the siida. For example, a herder might maintain a fence along the borders of his/her pasture to help themselves and their fellow siida members, incidentally benefitting neighbouring siidas. In the case of shared borders, neighbouring siidas would each have an incentive not to invest (i.e., to free-ride) if the other siida is likely to provision the good and they can reap the benefits without expending effort.

Borders to winter pastures can overlap and are permeable to a certain extent, depending on the needs of the reindeer – grazing rights in these patchy areas can shift to match herd size, for example, so siidas might tolerate a modicum of encroachment, especially in ‘emergency’ situations such as during bad winters (Marin and Bjørklund 2015). Herders are also sensitive to situations in which encroachment becomes a ‘shameful’ (Saami: *hæppat*) act of trespassing (Paine 1994). Herders will cooperate in a contingent and reciprocal manner, working with neighbouring siidas to separate herds when they become mixed, and ensuring against the degradation of lichen along shared migration corridors (Marin 2006). Indeed, the act of migration itself presents a coordination problem: the timing of migrations depends in part on the movements of neighbouring siidas (Tyler *et al.* 2007).

Government legislation and top-down management shape the social dilemmas facing pastoralists across scales from individuals to districts, notably the policy to reduce the number of reindeer on summer pastures by levelling quotas on districts. The quotas nominally aim to reduce the high levels of reindeer mortality observed in Finnmark, which might be due to density dependence (Tveraa *et al.* 2014), which negatively affects reindeer body condition and, hence, their reproduction (Bårdsen *et al.* 2010; Bårdsen and Tveraa 2012) and survival (especially during harsh winters: Tveraa *et al.* 2003; Bårdsen *et al.* 2011).

Districts, siidas and individual license owners are assigned absolute upper herd size limits based on average calf carcass weights (Reinert 2014), meaning that each herder is required to reduce their herds by a particular amount (apparently designed so that on average there is little to no change in the proportion of reindeer owned by any one herder compared to the rest of their siida). Quotas may be at odds with the competitive environment of northern Norway, in which herders might follow a strategy of herd accumulation in order to reduce risk (Næss and Bårdsen 2015). Thus a dilemma arises between pursuing the collective interest of acquiescing to the reindeer reduction policy and increasing one’s herd size as a means of ensuring long-term viability, the latter option being the rational, risk-reducing strategy (Næss and Bårdsen 2010, 2013).

Beginning in 1976, reindeer husbandry underwent a process of intensive commercialisation. This orientation towards the market incentivised herders to maximise meat production and thus their income (Hausner *et al.* 2011). Rather than simply maximising production, however, Saami pastoralists balance economic gains with the need to manage risk through increasing their herd sizes (Næss and Bårdsen 2015). An enforced shift away from customary land tenure towards ‘common’ or ‘open’ pastures—and, increasingly, privatised pastures—may be partly responsible for the overcrowding of reindeer in this area (Marin and Bjørklund 2015).

This form of rangeland fragmentation through privatisation might lead to increased degradation due to the higher concentration of reindeer and herders (Tømmervik *et al.* 2004) and might restrict herders’ mobility – potentially an important strategy for dealing with changing climatic conditions and especially extreme weather events (Tyler *et al.* 2007). Fragmentation and restricted mobility might also interfere with cooperative networks, which are important for continued viability in reindeer husbandry (Næss *et al.* 2010; Næss 2012).

### Testing Cooperation in the Field with Public Goods Games

A common tool for understanding the dynamics of cooperative behaviours in groups is the public goods game (PGG). PGGs involve a group of players individually deciding how much of an endowment to contribute towards a public account. Donations to the group are multiplied, often by a constant factor. The increased sum is then shared equally among all group members regardless of their initial contributions. When the multiplier is less than the group size, contributors receive less in return than they contributed. The ratio of multiplier to group size is known as the marginal per-capita return rate (MPCR; Ledyard 1995). Donations when MPCR <1 do not maximise utility and are interpreted either as acts of altruism (Camerer 2013) or mistakes (Burton-Chellew and West 2013).

When the multiplier is held constant, group size becomes an important factor in an individual’s cooperative calculus. PGGs played in the field (as opposed to laboratories) have found that larger groups can be more (Zhang and Zhu 2011) or less cooperative (Soetevent 2005) and this effect may not be linear (Yang *et al.* 2013). However, evidence for the importance of group size is equivocal; a meta-analysis of public goods games played across 18 countries found that group size (and thus MPCR) did not have a statistically significant effect (Balliet and Lange 2013). Factors such as participants’ ages, group composition, dispersal patterns, and social context are also likely to affect cooperation in groups (Balliet *et al.* 2011; Lamba and Mace 2011; Gerkey 2013; Waring and Bell 2013; Wu *et al.* 2015).

Here, we use an experimental public goods game to investigate how Saami reindeer herders in the county of Finnmark, northern Norway, respond to social dilemmas. Saami pastoralists work in cooperative groups of both kin and non-kin, and these groups cooperate and conflict to varying extents (Paine 1994; Thomas *et al.* 2015). Herding groups contain varying numbers of people and thus we expect to find differing levels of cooperation across groups.

Our study takes advantage of natural variations of group size within a single population where the groups are important hubs of cooperation. Given the evidence that group size affects levels of cooperation, we investigate how varying marginal per-capita return rates—as a function of group size—affects donations in a public goods game. This work thus departs from the usual structure of public goods games in that we did not artificially limit the number of participants in the groups but rather used real-world group sizes.

### Predictions

Much theoretical and experimental work has shown that people have strong preferences for cooperating with members of an ‘in-group’ (such as their siida) to the exclusion—and sometimes the detriment—of ‘out-groups’ (other siidas in the district) (Hewstone *et al.* 2002; Silva and Mace 2014). Whereas herders work with members of their siida on a regular basis, between-siida cooperation is more facultative. District-level cooperation is likely to be lower compared to within-siida cooperation, since the district is a large administrative area rather than a salient cooperative unit. (Note that this pattern of multiple siidas within a district is specific to our study site. In other parts of Norway, most districts contain only one summer siida.) Therefore, we expect donations to the siida PGG to be substantially larger than donations to the district PGG.

Following the theoretical models of how marginal incentives affect cooperation (Ledyard 1995), we predict that donations will increase as MPCR increases. Similarly, from kin selection theory, we predict that members of more closely related siidas will donate more compared to less-related siidas. Unlike theoretical models, but in keeping with observed behaviour in the laboratory and in the field (e.g., Cardenas and Carpenter 2008), we also predict that the majority of participants will donate non-zero amounts even though, by design, MPCR will always be less than one, thus creating a social dilemma in which the economically rational strategy would be to donate nothing.

### Study Area and Data Collection

Our study site was in a single reindeer herding district (District 16) in the county of Finnmark, Norway (see Thomas *et al.* 2015). In July – August 2013, the first author and a field assistant interviewed 30 licensed herd owners (42.3 % of all licensed herd owners in this district) belonging to all nine summer siidas in the district.

Participants played two public goods games then answered a quantitative survey (available on request). Self-reported cooperation was calculated by totalling the frequencies that each herder participated in eight communal activities, each measured on a 7-point Likert scale, ranging from ‘never’ (coded as 1) to ‘every day’ (coded as 7), for their own siida (within-siida cooperation) and on behalf of other siidas (between-siida cooperation). The activities were: common herding tasks, finding lost reindeer, taking animals to slaughterhouses, repairing fences and corrals, repairing cabins, repairing vehicles, producing handcrafts (Saami: *duodji*), and miscellaneous activities specified by study participants (Fig. 2).

### Public Goods Games

Participants played two one-shot public goods games (PGGs), deciding how much of their endowment to donate to the public good and how much to keep for themselves. Before each PGG, herders received vouchers for five litres of petrol in one-litre denominations. At the time this study was conducted, 1 litre of petrol cost approximately NOK 15 (US$ 2.54). They could choose to donate any amount to the group pot or keep as much as they wanted for themselves.

The first PGG was conducted with all participants in one large district-level group, with donations to the public good eventually distributed equally among all 30 participants. After participants made their donation decision in the district-level PGG, they played a second PGG where the group was formed only of members of the participant’s summer siida; both games followed the same protocol.

The total amounts donated in each PGG were multiplied by a factor of 1.5. This number was chosen because for some siidas, especially the smallest (*n* = 2), we could not guarantee in advance we would be able to interview more than two herders: the minimum number required for a social dilemma to exist. We were only able to interview one person for three of the nine siidas. Marginal per-capita return rate was calculated by dividing the PGG multiplier (1.5) by the group size. Three marginal per-capita return rates were calculated for each siida: (i) based on the number of license owners in the siida at the time of study (MPCR_licensed_), (ii) based on the total number of herders in the siida (MPCR_all_) and (iii) based on the number of license owners in the siida who ended up taking part in the experiment (MPCR_participants_).

Participants were told their PGG groups would be formed of license owners from the district (for the first game) and license owners belonging to their summer siida (for the second game). Players did not know at the point of participating how many other people in their siida would end up taking part, beyond their own insights into who might be likely to participate given their past experiences of working with these people. While the final group size was not necessarily known to each player (or the experimenters) at the time of the experiments, we hypothesise that players would expect a certain number of participants—and, hence, expect a certain return rate—given their knowledge of and history with their siida. The variations of group size within our study site act as a form of quasi-natural experiment in which ‘expected MPCR’ varies by siida.

After both donation decisions, we asked respondents why they chose to give particular amounts. Reasons were given verbally in either Saami or Norwegian, recorded in Norwegian by the field assistant and translated into English by the field assistant and the first author. Game scripts are available on request.

### Kinship Data

Kinship data were collected in May 2014 detailing how each pair of license owners in the district was genealogically related. We linked license owners to their previously assigned ID numbers and calculated a coefficient of relatedness (*r*_*ij*_) for each pair of herders (*i, j*). Mean relatedness to the siida was calculated by averaging each herder’s coefficient of relatedness to their fellow siida members.

### Statistical Analysis

As the amount an individual contributes to the PGGs can be no larger than the cash equivalent of five litres of petrol, we used Tobit regressions to account for this right-censoring (Tobin 1958). In each analysis, we selected and used the most parsimonious model for inference based on the candidate models’ (Table 1) second-order Akaike Information Criterion (AICc) values (Burnham and Anderson 2002). In the Tobit regressions, model selection was used to determine whether our three measures of marginal per-capita return rate (MPCR_licensed_, MPCR_all_ and MPCR_participants_), mean relatedness to the siida or total cooperation score best predicts donations in the siida PGG (Table 1). Each candidate model represents a working hypothesis: for instance, our separation between linear and curvilinear effects of group size was implemented as two different models.

**Table 1:** Set of candidate models for estimating donations in the siida public goods game. Each model contains a single predictor (apart from the null model). See main text for descriptions of how the variables were operationalised

Candidate models
Null model
Total self-reported cooperation score
Marginal per-capita return rate based on number of license owners in the siida (MPCR_licensed_)
Marginal per-capita return rate based on total number of herders in the siida (MPCR_all_)
Marginal per-capita return rate based on number of siida’s license owners that took part in the experiment (MPCR_participants_)
Mean relatedness within siidas

All analyses were conducted in R 3.2 (R Core Team 2012), using the package ‘VGAM’ (Yee and Wild 1996) to fit Tobit models. Plots were drawn using ‘ggplot2’ (Wickham 2009) with the ‘wesanderson’ palette generator (Ram and Wickham 2015). Analysis code and data files are available from https://doi.org/10.5281/zenodo.153816.

## Results

### People Cooperate More with their Herding Group

**Fig. 1:**
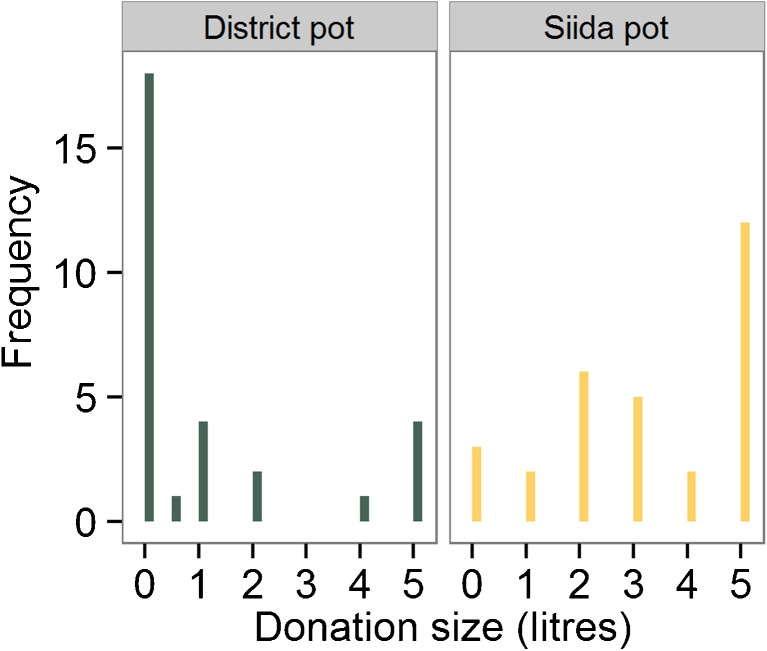
Distribution of donations in the district (*green*) and siida (*yellow*) public goods games. The median donation size in the district game was 0 l of petrol; the median donation in the siida game was 3 l

Participants donated more to their siida pot (median = 3 l of petrol) than to the district pot (median = 0 l; Fig. 1). Donations to the siida pot were significantly larger than donations to the district pot (two-tailed paired Wilcoxon test, *V* = 246, *p* < 0.001, 95 % CI [2.0, 4.0]). Eight people gave equal amounts to the district and their siida, while one person gave more to the district than to the siida.

Eighteen people gave nothing to the district and three people gave nothing to their siida. The same three people also donated nothing to the district. Five people donated 4 or more litres of petrol to the district pot. Of these, four donated all 5 l to their siida (the fifth donated 3 l). Of the four people donating all 5 l to the district, the primary source of income for two of them is through live reindeer sales, and for the other two through ‘other’ sources outside reindeer husbandry.

**Fig. 2:**
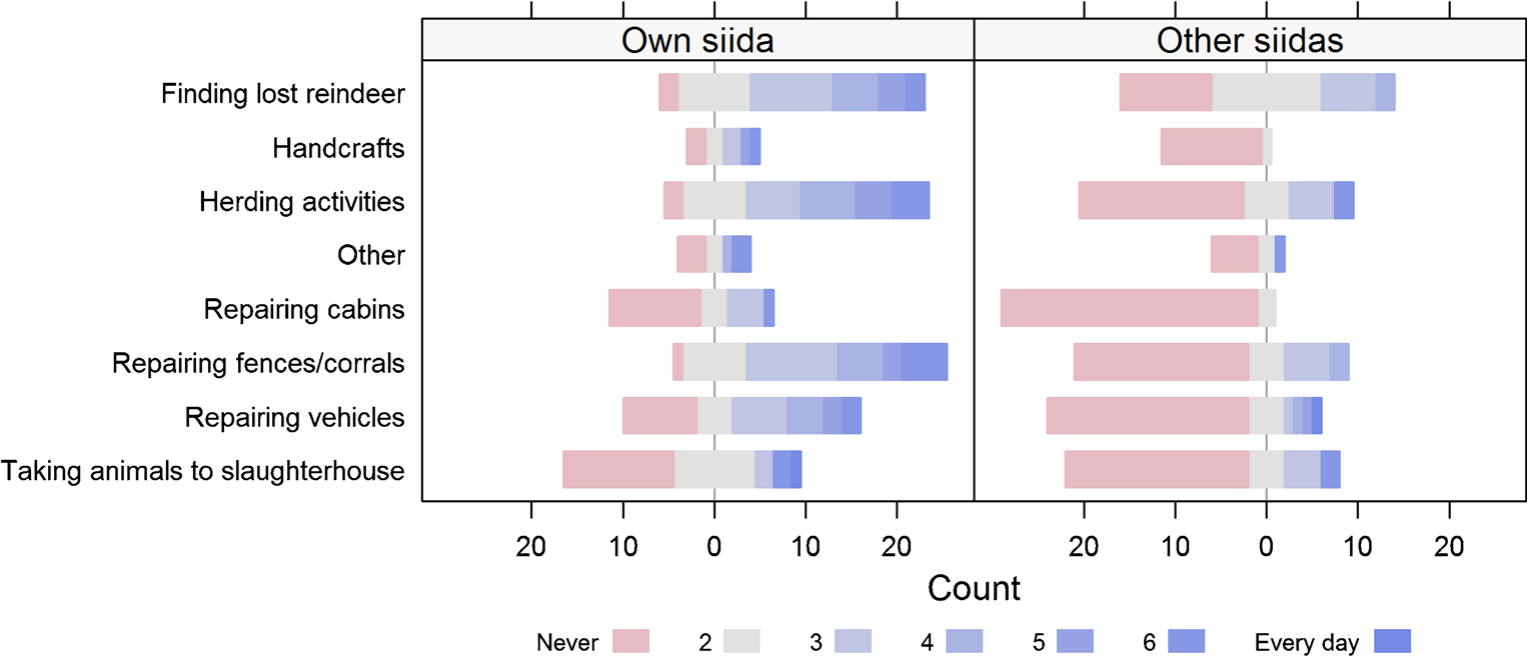
Self-reported frequencies for a range of cooperative activities that individuals take part in on behalf of their own summer siida (*left panel*) and for other siidas (*right panel*). Study participants were asked to report how often they had performed each activity on a scale of 1 (never) to 7 (every day) over the previous year. *Red bars* show the number of people who answered ‘never’; *darkening blue bars* show increasing frequencies of activity

**Fig. 3:**
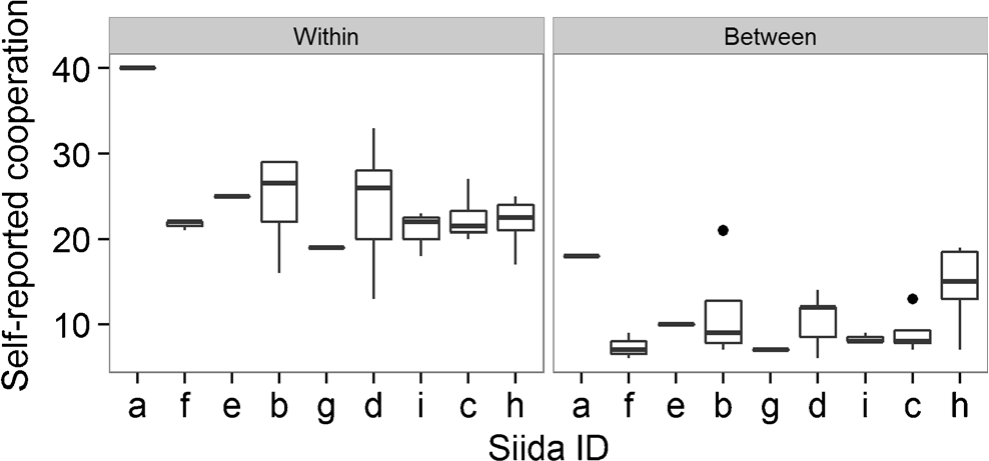
Total self-reported cooperation for each participant, clustered into siidas and split by whether cooperation was directed towards the participant’s own siida (*left panel*) or to other siidas (*right panel*). Siidas are ordered, left to right, in ascending order of size, based on number of license owners

Participants reported taking part in cooperative activities more frequently within their own summer siida than with other siidas (Fig. 2). For most questions, the majority of participants reported never taking part in the list of activities for other siidas. Total self-reported cooperation scores did not predict larger donations to the siida from more cooperative individuals (*B* =  − 0.126, *S*.*E*. = 0.115, *p* = 0.273). Similarly, herders who reported more cooperation with other siidas did not donate more to the district PGG (*B* = 0.032, *S*.*E*. = 0.272, *p* = 0.905). Siida size did not affect self-reported cooperation, either within or between siidas (Fig. 3). A participant’s mean relatedness to their siida did not predict contribution size (*B* = 6.011, *S*.*E*. = 4.313, *p* = 0.163).

### Marginal Incentives Matter

**Fig. 4:**
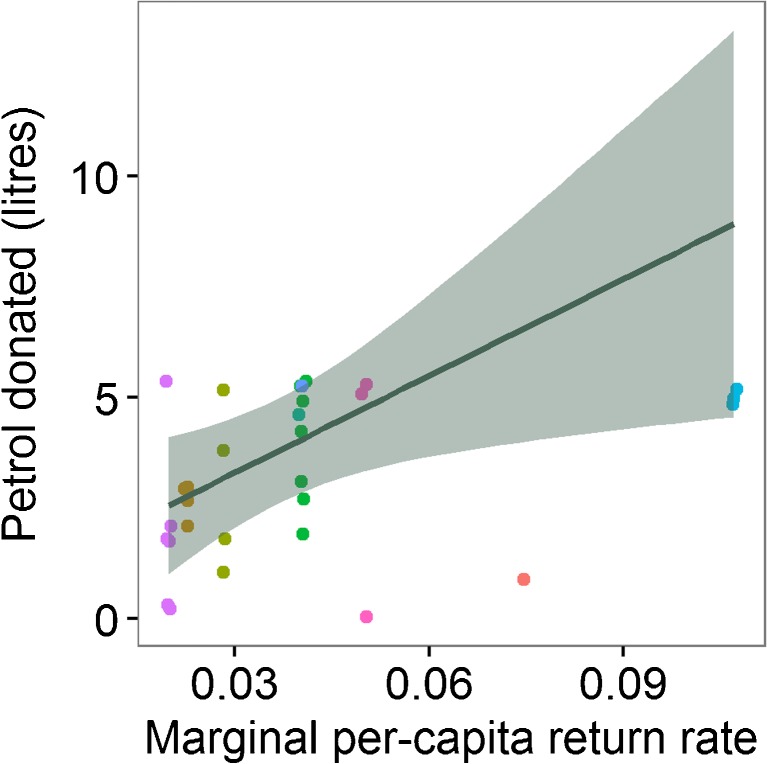
Marginal per-capita return rate predicts the size of donations to the summer siida public goods game (Table 3). Points show individual donations coloured by the summer siida of each herder. The line shows the fitted Tobit regression with 95 % confidence interval

Despite the district PGG’s marginal per-capita return rate being low (0.020), 12 people (40 %) donated at least 1 l of petrol to the district. In the siida PGG, MPCR depends on the number of herders in each group (Table 2). MPCR_all_ ranged from 0.020 in the largest siida (*n* = 76 people) to 0.107 in the smallest (*n* = 14 people), while MPCR_licensed_ was 0.083–0.750 and MPCR_participants_ was 0.214–1.500. MPCR_all_ was the best predictor of siida contributions (Table 3); increasing MPCR_all_ predicted larger donations to the siida (*B* = 72.820, *S*.*E*. = 30.373, *p* = 0.017; Fig. 4).

**Table 2:** Siida descriptive statistics, including the total number of herders in each siida, the number of herders owning licenses to herd reindeer, and the subset of license owners who took part in the public goods games. MPCR_all_, MPCR_licensed_ and MPCR_participants_ calculate the marginal per-capita return rates with respect to all herders, license owners only and the subset of license owners who participated. In all cases, the public goods multiplier was 1.5

Siida ID	Herders	License owners	PGG participants	MPCR_all_	MPCR_licensed_	MPCR_participants_
a	20	2	1	0.075	0.750	1.500
b	67	8	4	0.022	0.188	0.375
c	53	11	4	0.028	0.136	0.375
d	37	9	7	0.041	0.167	0.214
e	38	6	1	0.039	0.250	1.500
f	14	3	3	0.107	0.500	0.500
g	37	8	1	0.041	0.188	1.500
h	76	18	6	0.020	0.083	0.250
i	30	10	3	0.050	0.150	0.500

**Table 3:** Results from Tobit regressions estimating donations to the siida public goods game, including number of parameters (*K*), differences in AICc relative to the minimum in the set (ΔAICc), Akaike weights (*ω*_i_) and the log-likelihood of each model (LL). Marginal per-capita return rate given all siida members, not just license owners, (MPCR_all_) was the best predictor of donations

Model	*K*	ΔAICc	*ω*_i_	LL
MPCR_all_ (all herders in siida)	3	0.000	0.731	−48.495
Null model	2	4.454	0.079	−51.962
Relatedness	3	5.007	0.060	−50.999
MPCR_licensed_ (license owners only)	3	5.491	0.047	−51.241
Cooperation score	3	5.682	0.043	−51.336
MPCR_participants_ (no. participants in siida)	3	5.783	0.041	−51.387

The three people from the smallest siida seem to be driving the positive effect of MPCR_all_ (Fig. 4). Removing these three people from the model reduced the effect size and resulted in a non-significant effect (*B* = 47.948, *S*.*E*. = 44.564, *p* = 0.282).

Group size had a linear rather than curvilinear effect on PGG contributions (Table 4). For every additional person in the siida, there was an expected decrease in contributions of 0.079 l of petrol (S.E. = 0.027, *p* = 0.004). Restricting group size to include only license owners did not significantly predict donation size (results not shown).

**Table 4:** Results from Tobit regressions estimating the linear and curvilinear effects of group size on contributions to the siida public goods game. Columns report the number of parameters (*K*), differences in AICc relative to the minimum in the set (ΔAICc), Akaike weights (*ω*_i_) and the log-likelihood of each model (LL)

Model	*K*	ΔAICc	*ω*_i_	LL
Linear	3	0.000	0.751	−48.013
Curvilinear	4	2.661	0.199	−48.005
Null model	2	5.420	0.050	−51.962

### Reasons for Provisioning the Public Good

District donations were motivated by conditional cooperation, selfishness and reciprocity (or lack thereof). The largest donations to the district were given for reasons of collective action and conditional cooperation (Table 5). The majority of siida donation reasons were coded as due to collective action and the largest donations were given for reasons of collective action and reciprocity (Table 6).

**Table 5:** Coded reasons for donating to the district public goods game (or not), with number of participants giving that category of reason and total donations measured in litres. Donations categorised as ‘Other’ represent those that did not obviously fit into the theoretically derived categories. Full text of reasons is presented in Table S1

Category	No. donors	Total donations (litres)
Collective action	2	6
Conditional cooperation	6	11
Division of labour	1	0
Needs-based	2	2
Normative sharing	3	2.5
Prosocial	1	5
Reciprocity	6	0
Selfishness	7	1
Other	2	5
Total	30	32.5

**Table 6:** Coded reasons for donating to the siida public goods game (or not), with number of participants giving that category of reason and total donations measured in litres. Full text of reasons is presented in Table S2

Category	No. donors	Total donations (litres)
Collective action	14	48
Conditional cooperation	2	7
Division of labour	2	7
Kinship	2	5
Needs-based	1	2
Normative sharing	1	3
Prosocial	2	9
Reciprocity	2	10
Selfishness	4	6
Total	30	97

Tables S1 and S2 show the full stated reasons alongside herder IDs, so donations can be compared across tables. Three people gave the same reason for their siida donation as for their district donation (IDs 9, 16 and 82). Three of the four people donating all 5 l of petrol to the district did so because they knew the other people and wanted to support the district and/or reindeer husbandry. The fourth (ID 60) donated his entire endowment of petrol because he “can’t do much with 5 litres.” Two people (IDs 3 and 5) donated as a symbolic or normative gesture. Of the 18 people who donated nothing in the district PGG, 13 reasoned that they had no relationship with the district as a whole and would be unlikely to receive anything in return.

Overall, 21 people reported donating to their siida for reasons relating to cooperation, shared work and reciprocity. Two people donated in order to help younger herders. Herder 19 reported little cooperation in his summer siida, while herder 85 seemed to worry about whether other members of his siida would contribute to the PG.

There seemed to be some misunderstanding of the PGG. For example, herder 36’s response (Table S1) implied that he thought particular siidas would receive petrol from the PGG, as opposed to everybody receiving a share. Similarly, herder 97 donated only 1 l to his siida (Table S2) claiming that he and the only other license owner in the siida needed the petrol for themselves perhaps without realising they would be the ones receiving the pot (“Need it ourselves”) since they were the only eligible participants.

## Discussion

We investigated how cooperative behaviour within and between groups can solve social dilemmas. To do so, Saami reindeer herders played two public goods games (PGGs) – one where they could choose to donate petrol to their herding group (summer siida) and another where all participants were grouped within a larger administrative unit (the entire district). Participants donated larger amounts of petrol to their siida’s group pot than to the district, supporting the idea that the summer siida is an important locus of collective action. Contrary to expectations, increasing mean relatedness had no effect on cooperation within siidas, although this may be because we only measured relatedness between license owners rather than between all members of a siida. The natural variations in group size across siidas allowed us to investigate the effect of changing marginal per-capita return rate (MPCR) on PG donations. Larger MPCR—a feature of smaller groups—predicted an increase in donation size to the siida.

Participants donated to their group pots despite the return rate being less than one. Theoretically, individuals playing a strategy of pure self-interested utility-maximisation would donate nothing in this situation. Our results—in line with a number of laboratory and field studies—found that players donated despite the existence of a social dilemma. Our games assumed that herders would expect a particular return rate (either consciously or unconsciously calculated) depending on the number of other license owners in their siida and the likelihoods of their participation in our experiment. Although we did not explicitly test this assumption, the fact that marginal per-capita return rate with respect to the total number of herders in the siida predicted PG donations suggests that group size does play a role in cooperative decision-making. Whether this result generalises beyond our study population remains to be seen; meta-analyses of PGGs have found equivocal evidence for a group size effect depending on whether games were conducted in the field (Balliet and Lange 2013) or in laboratories (Zelmer 2003).

In order to gain a more detailed empirical understanding of how and why people contribute to public goods, future work should explicitly investigate participants’ expectations of returns on contributions and the extent to which pastoralists think strategically in terms of maximising their returns based on MPCR. It would also be valuable to understand the role that mechanisms such as reciprocity, reputation and social norms play in aligning interests to help groups solve their social dilemmas.

### Reported Cooperation and Reasons for Contributing to Public Goods Games

Self-reported cooperation, measured as the frequency that participants engaged in cooperative activities for their own as well as on behalf of other siidas, was not associated with donations to the siida’s public good. The lack of relationship implies either that self-reporting was not a useful metric of real-world cooperative behaviour or that game donations did not relate to real cooperation. The Likert scales employed here also have a particular shortcoming: they cannot capture rare events where herders turn up every time since these instances would be reported as a low Likert score. These issues may have ramifications for future field studies of cooperation that rely on survey methods and self-reports. In order to better understand social behaviours relevant to real-world situations, researchers should seek to combine these sociological methods with observational data and longer-term ethnography.

From the ex post facto reasons given by participants for their donation decisions, the majority donated for reasons pertaining to collective action (e.g., “We have the same job;” “It will come to good use for everybody in the siida;” “We work together”), including a normative approach to cooperation (“Everyone should participate”). A number of others reported reciprocity (“Someone in summer siida does something for me and I do something for them”). Conversely, those who donated nothing to their siida pot reported little cooperation in their siida or declared they needed a reason to donate.

While donating to their siida, people on the whole donated little to nothing to the district PG. Only four people gave their full endowment, whereas 18 donated nothing. Two people who donated their full endowments to the district seemed to reason in terms of large-scale collective action (“So reindeer herders keep the work going;” “Supporting [the district]”); another approached the dilemma in a more cautious, contingent manner (“If district asks he’ll give but if not, won’t give. Depends on situation”). Three participants donated 0.5–1 l as a normative signal of cooperation (“For good conscience;” “To show manners;” “A symbol of sharing”). One person stated that the endowment was too small (“Can’t do much with 5 [litres]”). Future work could involve more significant amounts of petrol, although the expensive nature of this field site might further limit the sample size attained. There were also hints towards demand sharing or needs-based cooperation regarding the district PGG (“If district asks he’ll give but if not, won’t give;” “Doesn’t give for no reason”). One participant stated after the interview that donation decisions may depend on the time of year; herders may need more petrol for themselves during summer, no matter how cooperative the siida.

### Cooperative Dynamics and their Implications for the Future of Saami Reindeer Husbandry

Understanding how Saami people solve their social dilemmas might have implications for the future of reindeer husbandry, especially given a background of shifting land tenure regimes, high density of reindeer, and climate change. Lab-in-the-field experiments such as our study can help to gain quantitative insights into the processes and patterns of cooperation among pastoralists as they face challenges from socio-ecological changes. Saami reindeer herders cooperate on a number of levels, from households up to districts. Each level also potentially sets the stage for conflicts. In our study district, cooperation was focussed within siidas and the majority of participants were unwilling to contribute to the district as a whole.

Not only is the scale at which cooperation occurs important but the number of potential cooperators also matters. It may be the case that smaller groups—who donated more to the siida’s PGG—have more opportunities to build trust through processes such as repeated reciprocal interactions, monitoring of behaviour, or interests that are more easily aligned. On the other hand, siidas with more license owners and other members tend to own more reindeer, meaning that they are better placed to hold larger or higher quality pastures (Paine 1994) as well as reducing risk and ensuring their long-term viability in reindeer husbandry (Næss and Bårdsen 2013).

We might expect that larger herds would encourage more cooperation as much as they result from efficient cooperation, since herding groups with more animals can potentially capture higher-quality land (Paine 1994). However, with the increasing privatisation of pastures, this may soon not be possible. On the other hand, more reindeer held by a siida might lead to more conflicts rather than cooperation because of the increased risk of reindeer mortality and worsening quality of reindeer as the overall number of animals in Finnmark has increased – a trend not simply due to climate change (Tveraa *et al.* 2013; Bårdsen *et al.* 2014).

Climate change is happening faster in the Arctic compared to the rest of the world, increasing the likelihood of extreme weather events (IPCC 2014). These events are likely to interact with population density, which negatively impacts reindeer body mass, reducing their survival and reproductive rates (Tveraa *et al.* 2003; Bårdsen *et al.* 2010, 2011; Bårdsen and Tveraa 2012). Therefore, herd accumulation alone might be a suboptimal strategy, despite being the dominant (and perhaps only viable) strategy in this area (Næss and Bårdsen 2013).

Ties to the land were once formed around customary access rights operating in a system of sequential usufruct (Reinert 2006). Increasingly privatised land-use rights are associated with an increased prevalence of fences in Finnmark (Kelman and Næss 2013). Fencing, in turn, might potentially limit access to migration corridors in the common spring/autumn areas (Marin 2006) and mobility more generally; a strategy of walking away from uncooperative others has been shown theoretically to foster cooperative outcomes (Aktipis 2011; Lewis *et al.* 2014). Herders in Finnmark emphasise the need for secure but flexible access to resources as opposed to land tenure reform per se (Marin 2006).

We plan to address in future work how changing climatic conditions and land-use rights are affecting mobility and social organisation in Finnmark. Saami herders have reported that communication and reciprocal cooperation are strong on the summer pastures in Finnmark, but that trust is lacking on winter pastures (Hausner *et al.* 2012). While this study focussed on cooperation within and between summer siidas, future work should investigate cooperative and competitive behaviours in winter siidas, where land tenure changes may create barriers to mobility and resource-access, and so potentially prove detrimental to a cooperative reindeer husbandry.

## Electronic supplementary material

Below is the link to the electronic supplementary material.

ESM 1 (DOCX 19.8 kb)
