# Supplementary material for: Smaller Saami Herding Groups Cooperate More in a Public Goods Experiment
Source: Hum Ecol Interdiscip J. 2016 Sep 19;44(5):633–42. doi: 10.1007/s10745-016-9848-3 (PMC5099356; doi:10.1007/s10745-016-9848-3)
Supplement: Supplementary file 1 — (DOCX 19.8 kb) [file 10745_2016_9848_MOESM1_ESM.docx]

# Supplementary Material for ‘Smaller Saami herding groups cooperate more in a public goods experiment’

Table S1: Reasons for donating to the district public goods game (or not) and donation size. Reasons were translated from the participants’ Norwegian or Saami responses. Categories match those summarised in Table 5.

| **ID** | **Donation reason** | **Donation (litres)** | **Category** |
| --- | --- | --- | --- |
| 1 | So reindeer herders keep the work going | 5 | Collective action |
| 2 | Supporting 16D | 5 | Prosocial |
| 60 | Can't do much with 5L | 5 | Other |
| 73 | People I know | 5 | Conditional cooperation |
| 36 | Those siidas who receive should keep their own animals at their own place to avoid herds mixing | 4 | Conditional cooperation |
| 16 | If district asks he'll give but if not, won't give. Depends on situation. | 2 | Needs-based |
| 85 | It depends on the time of year. It can be used for everything | 2 | Conditional cooperation |
| 5 | For good conscience | 1 | Normative sharing |
| 26 | It's necessary for people to drive | 1 | Collective action |
| 27 | For herding my reindeer | 1 | Selfishness |
| 49 | To show manners. If they [the district] really need it | 1 | Normative sharing |
| 3 | [Donation is] A symbol of sharing | 0.5 | Normative sharing |
| 9 | Has worked enough for free. [District] gets paid by the state. Have no relationship [with the district] | 0 | Selfishness |
| 18 | Not herder's choice to have one big district. Giving nothing | 0 | Selfishness |
| 19 | Give nothing back. Needs it himself | 0 | Reciprocity |
| 34 | Don't get anything back | 0 | Reciprocity |
| 35 | They [the district] don't need it | 0 | Needs-based |
| 40 | Doesn't give any fuel because district are not doing anything useful for him | 0 | Reciprocity |
| 46 | They are such a small siida it will have no influence | 0 | Conditional cooperation |
| 64 | Gets nothing and gives nothing | 0 | Reciprocity |
| 70 | They [the district] don't need it and they don't drive for me. They get money from the state | 0 | Reciprocity |
| 72 | Doesn't want to share | 0 | Selfishness |
| 74 | Because so many [people are] sharing the litres and challenging for a few litres | 0 | Conditional cooperation |
| 75 | Not sharing with district 16 | 0 | Selfishness |
| 76 | Don't know | 0 | Other |
| 77 | [The district] doesn't have any responsibility for driving. It is given to each siida | 0 | Division of labour |
| 82 | Doesn't give for no reason | 0 | Conditional cooperation |
| 86 | I need it myself. [The district] is too big for sharing | 0 | Selfishness |
| 97 | Needs it himself | 0 | Selfishness |
| 100 | Why give when I won't get anything back | 0 | Reciprocity |

Table S2: Reasons for donating to the siida public goods game (or not) and donation size. Reasons were translated from the participants’ Norwegian or Saami responses. Categories match those summarised in Table 6.

| **ID** | **Donation reason** | **Donation (litres)** | **Category** |
| --- | --- | --- | --- |
| 2 | Giving to siida. Working together | 5 | Collective action |
| 3 | Close family and they will have good use of it | 5 | Kinship |
| 18 | We have the same job | 5 | Collective action |
| 34 | It will be used for the purpose that I have needs for | 5 | Selfishness |
| 35 | It will come to good use for everybody in the siida | 5 | Prosocial |
| 36 | We are only 3. We have a good infrastructure. We are using the petrol together after a meeting about scheduling work. | 5 | Collective action |
| 49 | It's demanding to drive, so giving to younger herders because they have good health and agility | 5 | Division of labour |
| 60 | Someone in summer siida does something for me and I do something for them | 5 | Reciprocity |
| 64 | Gets it back anyway | 5 | Reciprocity |
| 70 | If I'm not there myself, the others can use it | 5 | Collective action |
| 73 | I also have a use for this | 5 | Conditional cooperation |
| 76 | Work together anyway | 5 | Collective action |
| 27 | Wants cooperation | 4 | Prosocial |
| 77 | We are often 5 who are driving | 4 | Collective action |
| 1 | They have the same herd and the same work | 3 | Collective action |
| 26 | Everyone should participate | 3 | Normative sharing |
| 46 | When we see all of the work for example in autumn is a lot of work | 3 | Collective action |
| 74 | Keeping work together (cooperating) | 3 | Collective action |
| 75 | We work together | 3 | Collective action |
| 5 | For the young people, for better yield | 2 | Division of labour |
| 16 | If district asks he'll give but if not, won't give. Depends on situation. | 2 | Needs-based |
| 40 | For cooperating and working against the predators | 2 | Collective action |
| 72 | Even/equal sharing | 2 | Collective action |
| 85 | If I take everything for myself it'll be used for driving the herd. If I give everything, will they give back? | 2 | Conditional cooperation |
| 100 | It comes to good use for everyone | 2 | Collective action |
| 86 | So the job will be done | 1 | Collective action |
| 97 | We are 2. Need it ourselves | 1 | Selfishness |
| 9 | Has worked enough for free. [District] gets paid by the state. Have no relationship | 0 | Selfishness |
| 19 | Little cooperation in summer siida. We (the family) drive for the most part | 0 | Kinship |
| 82 | Doesn't give for no reason | 0 | Selfishness |
